# Harnessing Oxylipins and Inflammation Modulation for Prevention and Treatment of Colorectal Cancer

**DOI:** 10.3390/ijms25105408

**Published:** 2024-05-15

**Authors:** Julius Gretschel, Racha El Hage, Ruirui Wang, Yifang Chen, Anne Pietzner, Andreas Loew, Can G. Leineweber, Jonas Wördemann, Nadine Rohwer, Karsten H. Weylandt, Christoph Schmöcker

**Affiliations:** 1 Medical Department B, Division of Hepatology, Gastroenterology, Oncology, Hematology, Palliative Care, Endocrinology and Diabetes, University Hospital Ruppin-Brandenburg, Brandenburg Medical School, 16816 Neuruppin, Germanyracha.elhage@mhb-fontane.de (R.E.H.); yifang.chen@mhb-fontane.de (Y.C.); anne.pietzner@mhb-fontane.de (A.P.); a.loew@ukrb.de (A.L.); can.leineweber@mhb-fontane.de (C.G.L.); jonas.woerdemann@mhb-fontane.de (J.W.); nadine.rohwer@mhb-fontane.de (N.R.); karsten.weylandt@mhb-fontane.de (K.H.W.); 2 Faculty of Health Sciences, Joint Faculty of the Brandenburg University of Technology Cottbus-Senftenberg, Brandenburg Medical School and University of Potsdam, 14476 Potsdam, Germany; 3 Department of Vascular Surgery, University Hospital Ruppin-Brandenburg, Brandenburg Medical School, Fehrbelliner Str. 38, 16816 Neuruppin, Germany; 4 Medical Department, Division of Psychosomatic Medicine, Campus Benjamin Franklin, Charité—Universitätsmedizin Berlin, Corporate Member of Freie Universität Berlin and Humboldt-Universität zu Berlin, 12203 Berlin, Germany; 5 Department of Molecular Toxicology, German Institute of Human Nutrition Potsdam-Rehbruecke, 14558 Nuthetal, Germany

**Keywords:** colorectal cancer, omega-3 PUFA, arachidonic acid, ASA, aspirin, statin, metformin, immune checkpoint inhibitors, prostaglandin E2

## Abstract

Colorectal cancer (CRC) is one of the most prevalent cancers worldwide, ranking as the third most malignant. The incidence of CRC has been increasing with time, and it is reported that Westernized diet and lifestyle play a significant role in its higher incidence and rapid progression. The intake of high amounts of omega-6 (*n* − 6) PUFAs and low levels of omega-3 (*n* − 3) PUFAs has an important role in chronic inflammation and cancer progression, which could be associated with the increase in CRC prevalence. Oxylipins generated from PUFAs are bioactive lipid mediators and have various functions, especially in inflammation and proliferation. Carcinogenesis is often a consequence of chronic inflammation, and evidence has shown the particular involvement of *n* − 6 PUFA arachidonic acid-derived oxylipins in CRC, which is further described in this review. A deeper understanding of the role and metabolism of PUFAs by their modifying enzymes, their pathways, and the corresponding oxylipins may allow us to identify new approaches to employ oxylipin-associated immunomodulation to enhance immunotherapy in cancer. This paper summarizes oxylipins identified in the context of the initiation, development, and metastasis of CRC. We further explore CRC chemo-prevention strategies that involve oxylipins as potential therapeutics.

## 1. Introduction

Colorectal cancer (CRC) is one of the most prevalent cancers, ranking as the third most common malignancy globally in 2018 and second in mortality rankings [1]. There have been more than 1.9 million new cases of CRC and 935,000 deaths in 2020. The increase in the incidence of CRC is considered a sign of economic and social development [2].

Common risk factors for CRC include smoking, drinking alcohol, low dietary fiber/calcium intake, increased red meat consumption, excess body weight, and physical inactivity [3]. Therefore, the change in lifestyle and dietary habits in most parts of the world toward Westernized diets and lifestyles probably contributes to the increased incidence of CRC. The majority of CRC entities are adenocarcinomas resulting from sporadic pathological epithelial damage and follow the adenoma–carcinoma sequence [4]. One of the well-known risk factors for the occurrence and development of CRC is a chronic inflammatory environment [5].

Eating habits around the world have changed dramatically in the Western world with increasing calorie and fat intake but also a predominance of the essential omega-6 polyunsaturated fatty acids (*n* − 6 PUFAs) in many diets, as compared with lower levels of the other essential fatty acid class, omega-3 (*n* − 3) PUFAs. These lifestyle changes could be an important reason for the increase in CRC prevalence. It is widely believed that for most of human history, our diet was based on an equal ratio of *n* − 6 to *n* − 3 PUFA uptake, while the present ratio is assumed to be approximately 15:1. This difference indicates a fundamental change [6].

## 2. PUFAs and Enzymatically Formed Oxylipins

**Figure 1:**
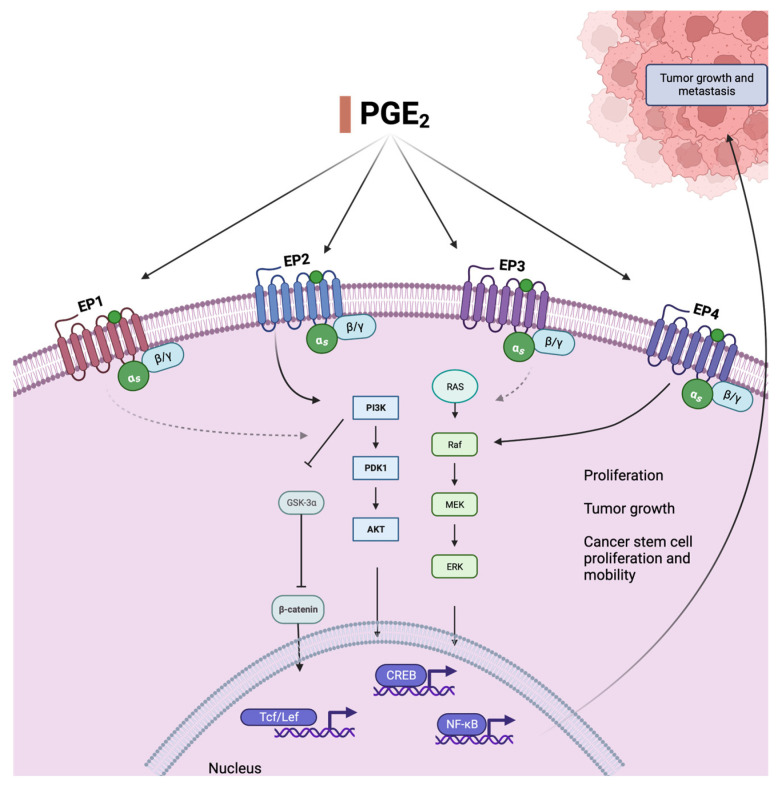
Prostaglandin E2, an oxylipin derived from arachidonic acid AA (20:4, *n* − 6), activates relevant cancer-promoting pathways. Binding to mostly two of its receptors (EP_2_ and EP_4_), it leads to the activation of Ras and Raf, upregulation of the PI3K/AKT pathway, and subsequent activation of pro-proliferative transcription factors. The receptors and pathways that are most relevant and best understood in this context are shown as full black arrows. The PI3K/AKT pathway is involved in cell survival, differentiation, and proliferation and additionally increases CRC-stem cell count and mobility, leading to metastasis and drug resistance, through activation of NF-kB. This effect is also caused by MEK-associated ERK activation, which is mostly triggered by binding to EP_4_. PI3K = phosphatidylinositol-4,5-bisphosphate 3-kinase, PDK1 = pyruvate dehydrogenase kinase 1, AKT = protein-kinase B, MEK = mitogen-activated protein kinase, ERK = extracellular-signal-regulated kinase, NF-kB = nuclear factor kappa-B, Tcf/Lef = T cell factor/lymphoid enhancer factor family, GSKa = glycogen synthase kinase 3.

**Figure 2:**
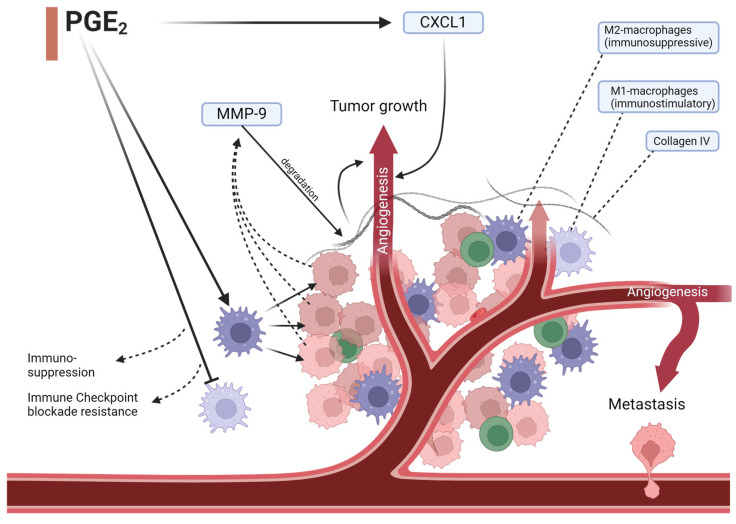
PGE_2_ impacts angiogenesis in multiple ways. It induces angiopoietin-2 expression, an important vascular growth factor, and impacts immune cell composition. Immunosuppressive macrophages get overexpressed and cause tumor cells to release increased amounts of MMP-9, which degrades collagen IV in the base membrane and the extracellular matrix enabling cancer-cell angiogenesis and metastasis. Angiogenesis allows for further cancer cell mobility and growth. Lower expression of immunostimulatory macrophages additionally hinders an appropriate immune response and restricts checkpoint inhibition therapy. MMP-9 = matrix metalloprotease-9, CXCL1 = CXC motif chemokine ligand 1.

Oxylipins are produced when omega-3 (*n* − 3) or omega-6 (*n* − 6) PUFAs are oxygenated by COX (cyclooxygenase), LOX (lipoxygenase), and CYP (cytochrome P450 monooxygenase) enzymes [7]. The process is mostly initiated by rising intercellular calcium levels that induce the cPLA2 (cytosolic phospholipase A2)-regulated release of PUFAs from the sn2-position of phospholipids in the cell membrane [8]. COXs are heme-containing enzymes with both oxygenase and peroxidase activities; they are able to convert free PUFAs into thromboxanes and 1-, 2-, 3-, and dihomo-2-series prostanoids, such as prostaglandin-D_2_ (PGD_2_) and prostaglandin-E_2_ (PGE_2_) [9]. PGE_2_ especially plays a key role in the context of CRC, is discussed extensively in Section 5, and is displayed both in Figure 1 and Figure 2.

**Figure 3:**
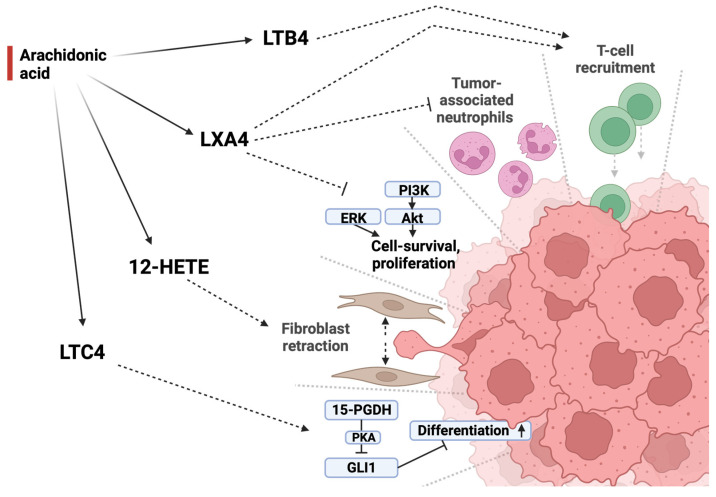
Different LOX-enzymes produce a variety of oxylipins from AA (20:4 *n* − 6) which exhibit different effects on tumor development. Lipoxin A4 acts in an antitumorigenic manner by downregulating both tumor-associated neutrophils as well as tumor-promoting pathways while promoting T-cell recruitment. This immunostimulatory effect is further supported by leukotriene B4. Leukotriene C4 acts in an antiproliferative manner by promoting cell differentiation through PKA-mediated inhibition of GLI1. 12-HETE, in contrast, promotes tumor development by causing fibroblasts in the tumor-adjacent stroma to retract, opening space for metastasis and tumor growth. LXB4 = lipoxin A4, LTB4 = leukotriene B4, 12-HETE = 12-hydroxyeicosatetraenoic acid, LTC4 = leukotriene C4, PI3K = phosphoinositid-3-Kinase, Akt = protein kinase B, ERK = extracellular-signal-regulated kinases, 15-PGDH = 15-prostaglandin dehydrogenase, PKA = protein kinase A, GLI1 = glioma-associated oncogene.

Another pathway is carried out by LOX enzymes, which starts with the production of hydroxy FAs (e.g., 5-HETE) and then subsequent modification into keto (e.g., oxo-ETE) or dihydroxy derivates (e.g., 5,15-diHETE). Activated LOX-5 catalyzes the formation of leukotrienes, and together with multiple consecutive LOX enzyme chain modifications, di- and tri-hydroxy FAs are created [10,11]. They include lipoxins, resolvins, protectins, and maresins. LOX-derived lipoxins like leukotrien-B_4_ (LTB_4_), lipoxin-A_4_ (LXA_4_), and 12-hydroxyeicosatetraenoic acid (12-HETE) are also featured in this review later on and are depicted in Figure 3.

Finally, CYP enzymes convert PUFAs in two main ways. Firstly, via epoxygenase, e.g., into epoxy-eicosatetraenoic acid (EpETE), epoxy-eicosatrienoic acid (EpETrE), and epoxy-docosapentaenoic acid (EpDPE), which are then remodeled by soluble epoxide hydrolase to become dihydroxy FAs such as dihydroxy-eicosatrienoic acid (DiHETrE). Secondly, via ω-hydroxylase, which creates, for instance, 20-hete, 19-hete, 20-hydroxyl leukotriene B4 (20-OH-LTB4), hydroxy-eicosapentaenoic acid (HEPE), HDoHE, and multiple ω-hydroxilated prostaglandins [11].

## 3. Increased Dietary Omega-3 PUFAs Might Lower Colorectal Cancer Risk

Given that the *n* − 6 PUFAs arachidonic acid (AA, 20:4 *n* − 6) is the precursor of the powerful often pro-inflammatory prostaglandin lipid mediators [12], the idea to change this disbalance by adding some *n* − 3 PUFAs is a straightforward concept in order to promote a less inflammatory and probably less CRC-prone nutrition environment.

Indeed, during the last three decades, data from both human and experimental studies have delivered evidence supporting the preventive use of *n* − 3 PUFA supplements in the context of CRC. A study published in 1993 investigated the effect of *n* − 3 PUFA oral supplementation with fish oil containing *n* − 3 PUFAs in a small group of twelve healthy people for 4 weeks and demonstrated reduced cell proliferation (used as a biomarker of decreased cancer risk) and decreased levels of PGE_2_ in rectal mucosa biopsy [13]. Another study, performed as a double-blind, placebo-controlled clinical study, was conducted in patients with sporadic adenomatous colorectal polyps treated with fish oil including eicosapentaenoic acid (EPA 20:5 *n* − 3, 4.1 g/day) and docosahexaenoic acid (DHA 22:6 *n* − 3, 3.6 g/day) for 12 weeks. The study also showed reduced proliferation in the upper part of colonic crypts [14].

Evidence from epidemiological studies supports these findings. The Physician’s Health Study recorded 500 male CRC patients over the course of 22 years and found an inverse correlation between fish and shellfish intake, or *n* − 3 PUFAs from other sources, and CRC risk [15]. Another large follow-up study collected data from 141,143 patients who were included in the Nurses’ Health Study 1, the Nurses’ Health Study 2, and the Health Professionals Follow-up Study, taking into consideration diet (assessing *n* − 3 PUFA intake through a validated food questionnaire), lifestyle, and medical information. Higher *n* − 3 PUFA uptake was connected with a lower risk of conventional adenomas (OR, 0.89; 95% CI, 0.84–0.95) and serrated polyps (OR, 0.90; 95% CI, 0.84–0.96) [16].

Several studies performed on mouse models of CRC underline the protective effect of *n* − 3 PUFAs. A study using human cancer xenografts in mice found that lower fat intake led to a decrease in tumor mass, which was even more pronounced (up to 90%) with *n* − 3 PUFA supplementation with lower levels of angiogenesis-associated gene expression in the colon tumors in the *n* − 3 PUFA-treated animals [17]. In the well-established azoxymethane (AOM)/dextran sodium sulfate (DSS) colon tumor mice model, animals fed with EPA (20:5 *n* − 3) showed decreased tumor incidence and size [18]. AOM and DSS were used for their ability to chemically induce DNA damage and cause colonic epithelial inflammation, leading to fast tumor formation. Results show that EPA (20:5 *n* − 3) decreased cell proliferation, PGE_2_ levels, and expression of nuclear β-catenin while increasing cell apoptosis in the model. These data match the results of our own study examining the effect of endogenously increased *n* − 3 PUFA levels in the fat-1 mouse model on CRC induction and development. The transgenic mice used in this study carry the fat-1 gene from the roundworm Caenorhabditis elegans, which encodes for a fatty acid *n* − 3 desaturase. Therefore, fat-1 mice can endogenously generate *n* − 3 PUFAs from *n* − 6 PUFAs, changing the *n* − 6/*n* − 3 PUFA ratio from values around 30/1 to approximately 1–5/1. Using the AOM and DSS model of CRC induction, we could demonstrate that these endogenously increased tissue levels of *n* − 3 PUFAs and almost balanced *n* − 6/*n* − 3- PUFA ratio lower the incidence and growth rate of colon tumors in fat—1 mice [19].

However, there is also inconsistent evidence, with some studies describing no or even negative effects of *n* − 3 PUFAs, as reviewed in [20], and whether the observed effects can be translated into practical recommendations for CRC prevention remains an open question. A currently ongoing study is assessing the effect of EPA (20:5 *n* − 3) as an adjunctive therapy in CRC patients with liver metastases undergoing partial liver resection with curative intent [21].

## 4. Nonsteroidal Anti-Inflammatory Drugs Prevent Colorectal Cancer

In 1988, a paper describing an inverse relation between acetylsalicylic acid (ASA) intake and CRC risk was published [22]. This was confirmed by multiple studies in the following decades, reporting CRC risk reduction rates ranging from 24 to 28% due to ASA use [23,24,25]. ASA and other nonsteroidal anti-inflammatory drugs (NSAIDs) inhibit both COX-1 and 2, thus reducing the amounts of prostaglandins produced. Specifically, the lowering of PGE_2_ levels, one of the main products of COX-2, is relevant as it has well-known pro-inflammatory and tumorigenic properties [26].

The protective effect is not limited to ASA but is also found with other COX-inhibiting NSAIDS, as demonstrated in a Danish-population-based case control study that saw a substantially decreased CRC risk in people using nonaspirin NSAIDs long term [27]. An Ohio study further specified these findings regarding different substances and their influence on CRC risk [28], with an odds ratio (OR) of 0.28 for ibuprofen or naproxen, and an OR of 0.28 for selective COX-2 inhibitors, showing that these substances have a substantial impact on CRC risk that is comparable to ASA (OR 0.33).

ASA, like many other NSAIDs, has a much higher affinity for COX-1 than 2 but is unique in its ability to disable them permanently and therefore inhibit blood clotting. Interestingly, this could prove helpful as well because of recent discoveries that described an increased platelet activation in CRC and linked it to several major steps of cancer progression. These include platelet-induced vessel and endothelial proliferation, cloaking of intravascular cancer cells, and even platelet stroma interactions that contribute to the inflammatory milieu [29].

Some studies indicate that acetylation by ASA changes COX-2 to no longer produce prostaglandins like PGE_2_ but anti-inflammatory lipid mediators such as aspirin-triggered lipoxins (derived from AA 20:4 *n* − 6) and resolvins (derived from *n* − 3 PUFAs) [30,31]. However, a recently published study did not see any evidence supporting the production of EPA-derived pro-resolving mediators [32]. Even individuals receiving both ASA and EPA supplements did not show any synthesis of ASA-triggered 15-epi-LXA_4_ or RvE1 in plasma or colon mucosa. This was also the case in an AOM/DSS mouse model study conducted by us in 2020, where ASA was administered in a dosage that is comparable to low-dose treatment in humans: although ASA exhibited all its established COX-inhibition-related effects such as attenuated platelet activation and decreased PGE_2_ formation, formation of ASA-triggered lipid mediators was not detectable [33].

Recommendations regarding regular ASA use for prevention of CRC are discussed controversially: while the United States Preventive Services Task Force issued a recommendation in its 2016 statement regarding its use in older adults [34], the update in 2022 did not uphold this [35], even though this is a matter of discussion and interpretation [36]. Regarding ASA treatment after diagnosis of CRC, survival benefit was only observed in patients with mutated PIK3CA (the phosphatidylinositol-4,5-bisphosphonate 3-kinase, catalytic subunit alpha polypeptide gene). PIK3CA mutations are present in 15–20% of CRC entities and play a key role in cancer development and progression, as we describe further in the following paragraphs [37]. Regular use of ASA increased CRC-specific and overall survival in these patients, while wild-type PIK3CA cancer patients did not benefit [38].

## 5. Lipid Mediators and Colorectal Cancer

PGE_2_ is the most important prostaglandin suppressed by NSAID treatment. It is derived from AA (through oxygenation by COX-1 or COX-2 and further modification by a PGE isomerase). Levels of PGE_2_ are increased in multiple cancer entities and promote carcinogenesis and metastasis in CRC. There are four different subtypes of PGE_2_ G-protein-coupled receptors: EP_1_, EP_2_, EP_3,_ and EP_4_ [39]. Activation of EP receptors 2 and 4 can upregulate the PI3K/Akt pathway (Figure 1), which is involved in cell proliferation, survival, and differentiation [40]. With activation of the PI3K/Akt pathway, β-catenin translocates into the nucleus, and COX-2 transcription and translation are triggered [41] (Figure 1), resulting in CRC cell migration and metastases [42].

A reduction in PGE_2_ might be even more relevant regarding its role in cancer stem cell (CSC) development and metastasis (Figure 1). A study published in 2015 found that PGE_2_ increased CSC numbers and migration leading to higher liver metastasis rates in mice. This effect could be decreased by a COX-2 blockade or knockdown of mediators like phosphoinositide 3-kinase (PI3K), EP_4_, or nuclear factor (NF)-κB. As such, PGE_2_ causes these effects through the EP_4_-PI3K and EP_4_-mitogen-activated protein kinase (MEK)-activated NF-κB [43].

Another significant step in tumor growth and metastasis that could be influenced by PGE_2_ is angiogenesis (Figure 2). It was demonstrated that PGE_2_ is able to increase angiogenesis due to C-X-C motif chemokine ligand 1 (CXCL1)-induction in CRC cells [44]. Additionally, it was shown to induce angiopoietin-2 expression in human endothelial cells and therefore increase an important vascular growth factor [45]. Moreover, PGE_2_ not only impacts tumor-associated signaling pathways but also immune cells in its microenvironment and the immune response toward cancer cells. When bound to EP_4,_ it induces the differentiation of immunosuppressive M2 macrophages while reducing immunostimulatory M1 macrophages (Figure 2). Not only does this inhibit an efficient reaction toward these cancer cells, but it also impairs possible immune-checkpoint inhibition treatment [46].

Another prostaglandin that has been implicated in CRC biology is PGJ_2_ in Kirsten rat sarcoma viral oncogene homolog (KRAS)-mutated CRC cells. 15-d-PGJ mediates the formation of stress granules that are an essential tool in KRAS-mutated cell stress resistance. This allows the tumor cells to survive even when physiological proliferative barriers are lost and sometimes even when chemotherapy is administered while continuing to multiply uncontrollably. Further knowledge concerning the mechanisms behind this stress resistance and possible ways of inhibition could therefore ameliorate the prognosis of the 35–45% of CRC patients with KRAS mutations [47,48]. In addition, elevated thromboxane A_2_ (TXA2) levels have also been established as a key player in CRC pathogenesis, and their inhibition could reduce malignant potential and slow down the spreading of CRC cells [49]. The effect of TXA_2_ is primarily achieved by activating platelets via G-protein-coupled receptors that lead to platelet aggregation and release of other mediators, promoting cell growth and migration [50]. Additionally, TXA_2_ might act in a pro-tumorigenic manner by upregulating Kv7.1 ionic potassium (K^+^) channels (also called KCNQ1 and KvLQT1) that participate in cell cycle progression and proliferation, via the cAMP pathway [51].

Oxylipins that are produced from AA (20:4 *n* − 6) through different LOX-enzymes also influence tumor development and progression in distinct and partly opposing ways, as shown in Figure 3 and presented in the following paragraphs. Enzymatic metabolism of AA (20:4 *n* − 6) by 12-LOX, for instance, generates 12-HETE. Colorectal adenocarcinoma cells secrete 12-HETE, causing the retraction of cancer-associated fibroblasts and thus opening up entry gates to the adjacent stroma [52]. A biomimetic of LXA_4,_ on the other hand, was found to inhibit the inflammatory state of the tumor microenvironment via the downregulation of ERK and the PI3K/AKT pathway in human dTHP-1 CRC cells. It furthermore decreased the level of tumor-associated neutrophils and myeloid-derived suppressor cells and increased T-cell recruitment intratumorally in a mouse xenograft colorectal carcinoma model [53,54]. Mast cells are another key player in immunity and have been suggested as a positive prognostic factor in CRC. Recent studies have indicated that Leukotriene B4 (LTB_4_) derived from these mast cells is essential for CD8^+^ recruitment, and mice lacking the LTB_4_ receptor had an increase in colon tumor progression and tumor-induced mortality [55]. Another LOX-derived oxylipin named LTC_4_ was found to induce the tumor suppressor 15-PGDH (15-hydroxyprostaglandin dehydrogenase), which leads to the downregulation of glioma-associated oncogene (GLI1) expression in a PKA (protein kinase A)-dependent manner, contributing to differentiation in CRC cells (Figure 3) [56]. 15-LOX-1 is downregulated in CRC cells, leading to lower levels of linoleic acid (LA) (18:2 *n* − 6)-derived 13-hydroxyoctadecadienoic acid (13-HODE) [57,58]. These lower levels of 13-HODE might contribute to tumor growth, as 13-HODE supplementation was shown in these studies to have an anti-proliferative effect on colon cancer cells.

Resolvin D1 (RvD1), derived from DHA (22:6 *n* − 3), has been studied regarding its effects on CRC and associated inflammation. It was reported that RvD1 possesses protective properties which are mediated through the blockade of IL-6 receptors, JAK2/STAT3, and following Cyclin D1 downregulation [59]. Additionally, a study demonstrated that RvD1 lowered the overexpression of c-Myc protein in HTC 116 human colon cancer cells through two separate mechanisms. First, it enhanced its ubiquitination and subsequent proteasomal degradation, and second, it inhibited its stabilization through extracellular-signal-regulated kinase-mediated phosphorylation by direct interaction with the ALX/FPR2 receptor [60].

**Figure 4:**
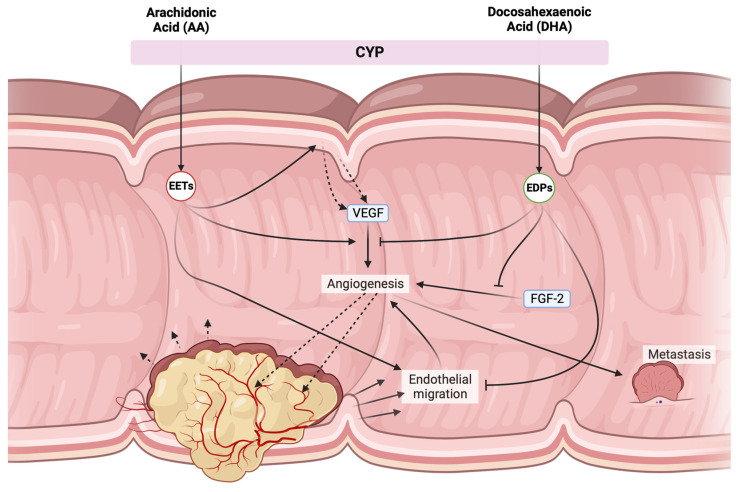
Opposing effects of CYP products derived from AA (20:4 *n* − 6) and DHA (22:6 *n* − 3). EETs mostly possess pro-tumorigenic effects, triggering local VEGF secretion and enhancing VEGF-receptor 2 expression, while also promoting endothelial migration allowing for further angiogenesis. Additionally, they might cause metastasis through VEGF release. EDPs suppress many of these effects by blocking both VEGF and FGF-2-dependent angiogenesis, as well as dampening endothelial cell migration. VEGF = vascular endothelial growth factor, CYP = cytochrome P450 enzymes, FGF2 = fibroblast growth factor 2, EETs = epoxyeicosatrienoic acids, EDPs = epoxyeicosatetraenoic acids.

Another DHA-derived lipid mediator important in the context of CRC is CYP P450-derived epoxydocosapentaenoic acids (EDPs). EDPs potently inhibit cancer growth, neovascularization, and metastasis both in vivo and in vitro [61], due to the inhibition of vascular endothelial growth factor (VEGF)- and fibroblast growth factor 2-induced angiogenesis (Figure 4). When combined with a low-dose inhibitor of the soluble epoxide hydrolase (sEH), thereby stabilizing the epoxy compounds, EDPs’ effects were further strengthened, leading to an approximately 70% decrease in tumor growth and metastasis [61]. This is in contrast to the effect of LA (18:2 *n* − 6)- and AA (20:4 *n* − 6)-derived epoxy compounds, which showed pro-tumorigenic effects in AOM/DSS-induced colon cancer in mice [62,63]. As depicted in Figure 4, these effects were caused by an increase in local VEGF secretion and receptors coupled with enhanced endothelial migration.

## 6. Chemoprevention Strategies in Colorectal Cancer—Beyond NSAIDS

With commonly used NSAIDs, and particularly ASA, the concept of CRC chemoprevention has been proven, with the ongoing discussion of whether ASA administration might even become a widespread recommendation to lower CRC risk. As this NSAID effect is due to the modulation of lipid mediators, particularly PGE_2_ suppression, we questioned whether other commonly used drugs that have been implicated in the chemoprevention of CRC also have effects on lipid mediator formation.

Statins are another widely used group of substances that have been analyzed concerning their potential as cancer chemoprevention agents. As much as 35% of people in the US were taking them in 2018–2019 for their lipid-lowering properties that help prevent cardiovascular disease [64]. A case control study by Poynter et al. that included nearly 4.000 subjects showed an astonishing 43% reduction in CRC risk associated with 5 or more years of self-reported statin intake [65]. A retrospective cohort study of US veterans reported a risk reduction of 35% in a dose-dependent manner [66]. It has also been demonstrated that statins hindered growth and promoted apoptosis in human CRC cell lines [67]. Statins competitively inhibit the 3-hydroxy-3-methyl-glutaryl-coenzyme A (HMG-CoA) reductase, the rate-limiting enzyme of the mevalonate pathway. This leads to a relative cholesterol deficiency inside the cell, encouraging it to produce higher quantities of LDL receptors, thus taking more LDL out of the bloodstream. However, the HMG-CoA reductase inhibition affects not just cholesterol levels but also other intermediates of the mevalonate pathway, including farnesyl pyrophosphate (FPP) and geranylgeranyl pyrophosphate (GGPP) [68]. Their inhibition is relevant as they are needed for post-translational modification (isoprenylation) and activation of many different cellular proteins [69]. The important ones are Ras and Rho, two small GTPases that are essential parts of signaling pathways for cell growth, gene expression, apoptosis and inflammation [70,71]. It has been shown that the dysregulation of the mevalonate pathway is able to drive cancer development [72].

Atorvastatin was shown to lower pro-inflammatory markers [73], and statins were shown to reduce CRP levels and pro-inflammatory cytokines [74] while upregulating CD4+ and CD25+ regulatory cells [75]. Additionally, atorvastatin inhibits platelet-dependent COX-2 expression in endothelial cells in a CD40-dependent manner [76]. This is relevant as platelets can enhance chemotaxis of inflammatory cells and vascular wall inflammation by releasing pro-inflammatory mediators [77], and inflammation modulation by statins might not only be beneficial for the treatment of the inflammatory process atherosclerosis [75] but might also influence carcinogenesis-associated inflammatory aspects. Furthermore, antioxidant effects [78] and effects on angiogenesis [79] and cell adhesion [80] have been described.

Interestingly, there is increasing evidence that statins increase AA (20:4 *n* − 6)-derived oxylipins. Several studies demonstrate that statins appear to increase the enzymatic activity of fatty acid desaturase 1 (FADS1), the rate-limiting enzyme of C20 PUFA synthesis from C18 precursors, such as the conversion from LA to AA c [81,82,83]. Indeed, we also observed increased levels of several AA-derived oxylipins, most notably PGD_2_ in the colon tissue of individuals receiving statin treatment compared with untreated subjects or subjects on ASA medication [84]. Interestingly, statins and ASA co-treatment blunted this increase. While most AA-derived oxylipins such as PGE_2_ are mostly pro-inflammatory (*n* − 6 pro-inflammatory oxylipins), PGD_2_ has been shown to hinder tumor progression, which might explain this apparent contradiction [85]. The impact of statins on AA (20:4 *n* − 6) metabolism and their anti-proliferative and inflammation-dampening role described above is still not completely understood.

Metformin is a frequently used drug for the therapy of type 2 diabetes, which is a risk factor for CRC [86]. A recent meta-analysis including 58 studies concluded that metformin users had a substantially lower incidence of colon adenomas, advanced adenomas, and CRC [87]. Additionally, it was shown that outcomes of metastatic CRC patients were improved, and overall survival, as well as CRC-specific survival, increased in those taking metformin. This was also confirmed in a Korean national cohort study including more than 320,000 people [88]. Participants with type-2 diabetes receiving metformin treatment had a lower risk of developing CRC not only compared with other type 2 diabetics but also compared with people without the condition. These findings highlight metformin’s effects that appear to surpass the dampening of a diabetes-associated CRC risk increase. One mechanism that might explain how this effect is mediated was explored in a bladder cancer model in which metformin was able to inhibit stem cell multiplication by reducing COX-2-mediated PGE_2_ and following STAT3 activation both in mouse bladder cancer and bladder cancer cell lines [89]. Changes in AA (20:4 *n* − 6)-derived oxylipins were also detected in studies investigating metabolic changes in healthy subjects receiving metformin [90,91]. Another study demonstrated that metformin was able to reduce EET formation [92], possibly by binding to the active site heme of CYP3A4, depleting cancer cells of AA-derived EETs and their pro-tumorigenic effects, such as angiogenesis and mTOR signaling [93].

## 7. Concomitant Medications and Immune Therapy

Given the importance of immune-modulating effects described for the chemopreventive approaches in CRC, often involving oxylipin pathways and particularly the PGE_2_ pathway, it is tempting to assume that these effects could become more important also in the context of prevention and treatment of cancer with advances in immune checkpoint inhibitor (ICI) therapy. A 2020 study was able to demonstrate that by reducing PGE_2_’s effect on immune cell (Figure 2) responsiveness to anti-PD-1, therapy in mice could be ameliorated [46]. This was achieved by blocking EP_4_ which led to a decreased function of immunosuppressive cells and enhanced cytotoxic T-cell-mediated tumor elimination. Tumor progression was reduced and survival in treated mice was prolonged. This shows how our understanding of oxylipins could help develop strategies to enhance already existing immunological therapy concepts.

When looking at the previously discussed chemoprevention agents, ASA treatment in particular might have synergistic effects in the context of immune checkpoint inhibitor therapy. COX activity was proposed as a main factor causing immune suppression across species, and when reduced, left mice CRC cells more susceptible to immune control [94]. This effect was again traced back to PGE_2_, as its immunosuppressive effects were essential for mutant BRAF mouse melanoma cells to grow in immune-competent organisms. Other substances similarly show promising results in animal studies. Metformin increased sensitivity towards PD-1 inhibition and increased CD8+ T-cell infiltration in lung cancer [95]. Additionally, the degradation of PD-L1 in mice breast cancer has been described [96]. Statins were shown to have comparable properties, as they were able to enhance T-cell activity and reduce PD-L1 expression in breast cancer. Atorvastatin supported the effect of a co-administered anti-PD-L1 therapy in vitro [97]. Recently, an experimental study was able to show that in animal models with dietary omega-3 (*n* − 3) polyunsaturated fatty acid supplementation and increases in their CYP-epoxyeicosanoids by pharmacologic inhibition of the sEH, the anti-tumor activity of ICI is enhanced [98].

While all of these results seem promising, data from human studies are currently often inconclusive: in contrast to the effect of steroids, ref. [99] baseline statin, and ASA (and beta-blocker), medications were associated with better tumor response to ICI treatment. Another study published in 2021 also found beneficial effects of low-dose ASA and/or statin administration in addition to ICI therapy [100]. However, a beneficial effect was not seen with COX inhibitors and ICI in another study of lung cancer [101]. Another recent study found higher rates of immune-adverse events with ASA treatment in patients undergoing ICI treatment [102]. On the other hand, there are also initial promising data with metformin plus ICI in melanoma [103], as well as metformin plus ICI in lung cancer [104,105].

This difference could be explained in part due to many different co-medications, making the elucidation of modulating effects difficult. Specifically, many cancer patients receive steroids as part of their combination therapies, which are powerful immune-modulating compounds that might well blunt all other effects—and were shown to have a worse outcome in combination with ICI therapy [99].

## 8. Conclusions and Perspectives

In this comprehensive review, we examined the current understanding of CRC prevention strategies, with a primary focus on inflammation modulation, particularly through the manipulation of PUFA-derived oxylipin pathways. Central to our discussion is the modulation of the PGE_2_ signaling pathway. We also discuss several other oxylipin pathways that have shown promise in thwarting carcinogenesis. While both epidemiological studies and experimental evidence have suggested a lowered CRC risk with *n* − 3 PUFA supplementation and therefore a change in lipid mediator composition, the establishment of clear intake or supplementation recommendations remains elusive.

The effectiveness of NSAIDs, particularly ASA, in preventing CRC is well-supported by robust data. However, ongoing debates persist regarding the optimal recommendations for ASA use in CRC prevention. One of the major challenges lies in treating a large number of patients over extended periods to prevent relatively rare events, while also considering the potential risks of side effects associated with agents such as ASA, statins, or metformin. This complexity is further compounded in Western countries, where colonoscopy-based screening strategies are already firmly established as preventive measures. However, the insights gleaned from our exploration of oxylipin pathways offer intriguing possibilities not only in CRC prevention but also in addressing other malignancies. Many gastrointestinal cancers and other malignancies exhibit an inflammatory component in their tumorigenesis process, suggesting the potential applicability of these approaches across various cancer types.

Moreover, as the landscape of cancer treatment evolves, with immune checkpoint inhibition (ICI) assuming a central role, there emerges an opportunity to extend oxylipin concepts from prevention to treatment optimization. Understanding the immunomodulating effects of CYP-, LOX-, and COX-derived oxylipins can help to employ them to increase the effectiveness of these therapeutic strategies. Future endeavors could benefit from analyzing oxylipin pathways within the context of immune-modulation-based cancer therapies, thereby paving the way for novel approaches to treatment optimization.

## Data Availability

No new data were created or analyzed in this study. Data sharing is not applicable to this article.
